# Increasing the Efficiency of a National Laboratory Response to COVID-19: a Nationwide Multicenter Evaluation of 47 Commercial SARS-CoV-2 Immunoassays by 41 Laboratories

**DOI:** 10.1128/JCM.00767-21

**Published:** 2021-08-18

**Authors:** Maaike J. C. van den Beld, Jean-Luc Murk, Jan Kluytmans, Marion P. G. Koopmans, Johan Reimerink, Inge H. M. van Loo, Marjolijn C. A. Wegdam-Blans, Hans Zaaijer, Serology Workgroup for SARS-CoV-2, Corine GeurtsvanKessel, Chantal Reusken

**Affiliations:** a Centre for Infectious Disease Control, WHO COVID-19 Reference Laboratory, National Institute for Public Health and the Environment, Bilthoven, the Netherlands; b Microvida, Elisabeth-Tweesteden Hospital, Tilburg, the Netherlands; c Amphia Ziekenhuis Breda, Microvida Laboratory for Microbiology, Breda, the Netherlands; d Julius Center for Health Sciences and Primary Care, University Medical Center Utrecht, Utrecht University, Utrecht, the Netherlands; e Department of Viroscience, Erasmus MC, Rotterdam, the Netherlands; f Department of Medical Microbiology, Maastricht UMC+ and Care and Public Health Research Institute, Maastricht University, Maastricht, the Netherlands; g Department of Medical Microbiology, Laboratory for Pathology and Medical Microbiology (PAMM), Veldhoven, the Netherlands; h Sanquin Blood Supply Foundation, Amsterdam, the Netherlands; St. Jude Children's Research Hospital

**Keywords:** SARS-CoV-2, COVID-19, immunoassays, laboratory response, diagnostics, serology

## Abstract

In response to the worldwide pandemic of severe acute respiratory syndrome coronavirus 2 (SARS-CoV-2) and the subsequent antibody tests that flooded the market, a nationwide collaborative approach in the Netherlands was employed. Forty-one Dutch laboratories joined forces and shared their evaluation data to allow for the evaluation of a quantity of serological assays for SARS-CoV-2 that exceeds the capacity of each individual laboratory. As of April 2020, these performance data had been aggregated and shared in regularly updated reports with other laboratories, Dutch government, public health organizations, and the public. This frequently updated overview of assay performance increased the efficiency of our national laboratory response, supporting laboratories in their choice and implementation of assays. Aggregated performance data for 47 immunoassays for SARS-CoV-2 showed that none of the evaluated immunoassays that detect only IgM or IgA met the diagnostic criteria, indicating that they are not suitable for diagnosing acute infections. For the detection of IgG, only the Biozek Corona virus COVID rapid test, Euroimmun SARS-CoV-2 IgG, and Wantai SARS-CoV-2 antibody (Ab) ELISA met predefined performance criteria in hospitalized patients where samples were collected 14 days post-onset of symptoms (DPO), while for patients with mild or asymptomatic infections, only the Wantai SARS-CoV-2 Ab ELISA met the predefined performance criteria if samples were collected 14 days postonset. Here, we describe this unique nationwide collaboration during the onset of the COVID-19 pandemic; the collected data and their results are an example of what can be accomplished when forces are joined during a public health crisis.

## INTRODUCTION

In December 2019, patients with pneumonia and an infection with what was later identified as severe acute respiratory syndrome coronavirus 2 (SARS-CoV-2) were admitted in hospitals in Wuhan, China. The virus rapidly spread within China and across borders. As of 31 March 2021, 127,349,248 cases, including 2,787,593 deaths, have been reported to WHO (1).

The rapid global spread of SARS-CoV-2 demanded a cooperative, rapid, and efficient laboratory response worldwide. By 29 January 2020, 24 member states of the European Union, including the Netherlands, had molecular testing implemented in at least one laboratory, providing the basis for a large scale up in molecular testing capabilities throughout Europe in the following weeks (2). Although laboratories in Europe were highly efficient, a complication was that most initially relied on the same protocols and platforms (3), while shortages in high-quality supplies for diagnostic testing were building up (4, 5). These shortages even resulted in supranational inventories to identify critical issues in the supply chains and coordination of the procurement of supplies (6).

In March 2020, the Dutch government installed the National Test Capacity Coordination Structure (LCT) to monitor and ensure a sufficient and accurate test capacity across the nation (7). Although the primary focus and priority of the LCT was the molecular diagnosis of SARS-CoV-2, a Serology Taskforce was installed under the LCT at second instance when the offers for serological assays were building up and concerns arose about an overstrained immunoassay market. The taskforce consisted of 10 medical microbiology experts and was coordinated by the Institute for Public Health and the Environment (RIVM). The taskforce was requested to advise the LCT on the use of serology in general and of specific immunoassays in patient management and the control of the pandemic. The efforts of the Serology Taskforce consisted of monitoring the usefulness of serological testing in different patient populations and study designs, advising on national policy regarding employment of serology tests for mitigation strategies, and coordinating an efficient laboratory response in the Netherlands regarding the application of serological tests.

One of the needs identified was to provide rapid and evidence-based advice on the use of specific immunoassays to support laboratories in their choice of assay implementation and to support the LCT in guaranteeing access to those tests. The market for immunoassays is overwhelming, with a total of 605 commercialized immunoassays listed by the Foundation for Innovative New Diagnostics (FIND) in their SARS-CoV-2 diagnostic pipeline as of 31 March 2021 (8).

In the Netherlands, a nationwide effort was undertaken to collect, aggregate, and share evaluation data on immunoassays at a national level. Here, we describe the outcomes of our unique approach to a collaborative Dutch laboratory response to the SARS-CoV-2 pandemic which resulted in a multicenter evaluation of 47 commercial SARS-CoV-2 immunoassays. The outcomes of the assay evaluations by 41 laboratories are presented as an example of what can be accomplished by such a nationwide approach in which forces are joined to support international SARS-CoV-2 laboratories in informed decision-making on immunoassay implementation.

## MATERIALS AND METHODS

### Data collection and dissemination of results.

Inventories of ongoing immunoassay evaluations were carried out via the Dutch Society for Medical Microbiology (NVMM) (9). Starting 28 March 2020, weekly requests for data sharing were sent to members of the NVMM, consisting of 440 medical microbiologists, medical molecular microbiologists, and their trainees, employed by 1 of the approximately 50 registered Dutch Medical Microbiological laboratories. Data reported by laboratories that are ISO 15189:2012 (10) accredited with a flexible scope in the fields medical microbiology for codes MM.VID.15 or MM.VID.16 or medical immunology for codes MI.IFS.01, MI.IFS.02, or MI.IFS.04 were summarized and shared in regularly updated reports (https://www.nvmm.nl/vereniging/nieuws/update-taskforce-serologie-15-juli-2020/). All laboratories voluntarily contributed data, accompanied by available metadata such as date of onset, sampling date, disease severity, and age of patients, and their permission for sharing their aggregated data was obtained.

**FIG 1:**
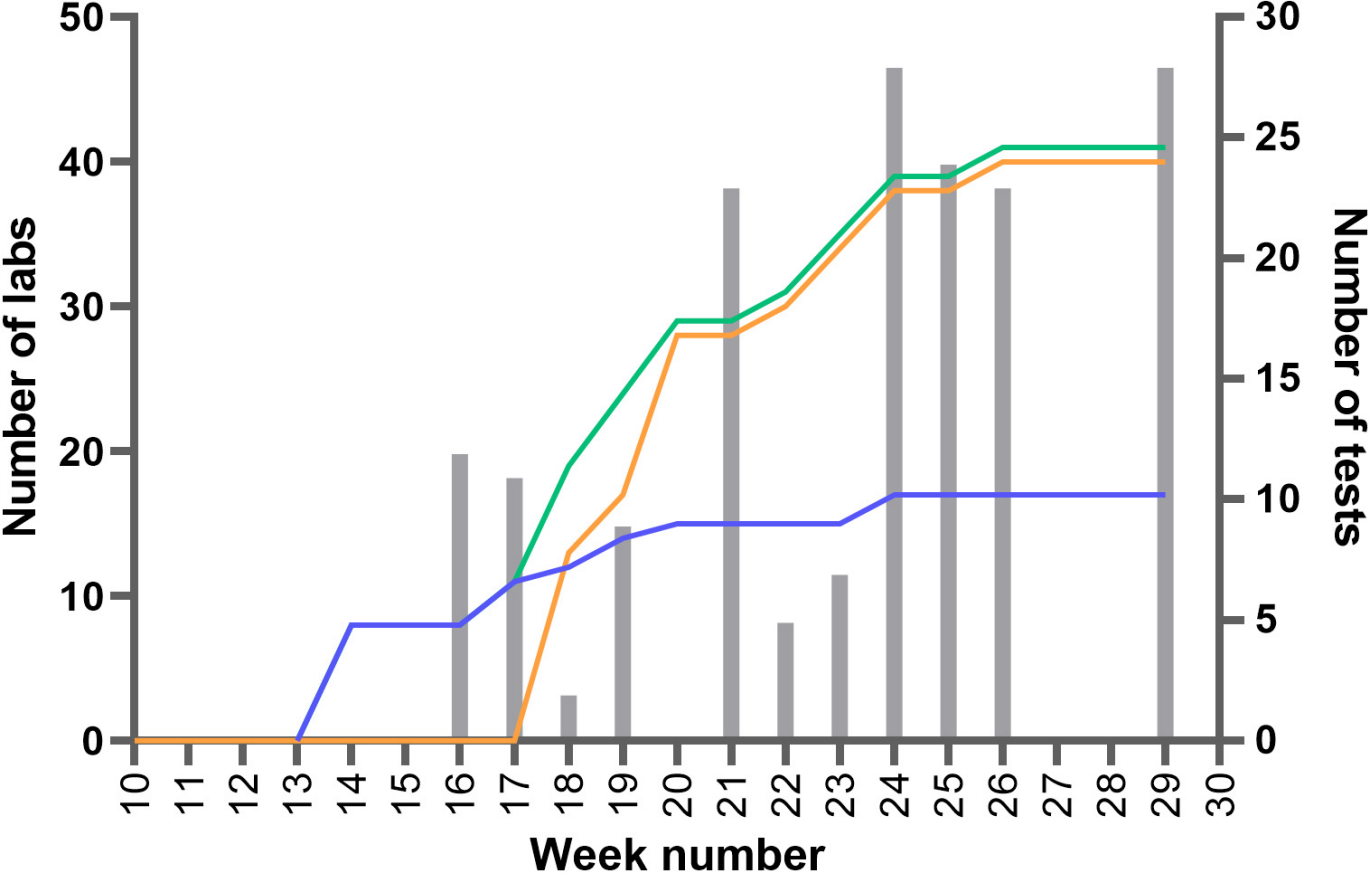
Numbers of contributing labs and number of tests with newly shared data over time. Left *y* axis: blue line, labs that contribute data for POC tests; orange line, labs that contribute data for ELISA/autoanalyzers; green line, contributing labs in total. Right *y* axis: gray bars, the number of tests for which new data were published.

By the end of July 2020, 41 laboratories were contributing to the rapid sharing of aggregated data across laboratories (Fig. 1). In the period from 13 April 2020 to 17 July 2020, 16 reports were shared. By then, information had been collected and shared for 47 different immunoassays. Collection and sharing of data in updated reports was done at an almost weekly basis to ensure a rapid access to new relevant data by (inter)national laboratories and other stakeholders.

The reports drafted between 13 April and 5 May 2020 were privately shared with Dutch medical microbiologists via the NVMM; the national Outbreak Management Team; the Dutch ministry of Public Health, Welfare and Sport; the European Centre for Disease Prevention and Control (ECDC); and the WHO along with a summary that was publicly available. During this period, other stakeholders, e.g., physicians, laboratory managers, public health experts, and manufacturers, expressed their interest in the full reports. With permission of the contributing laboratories, full reports were made public as of 19 May 2020. The most recent version of the reports are available on the websites of the NVMM (11) and the Institute for Public Health and the Environment (RIVM) (12). Reports as of 2 July were disseminated in English upon many requests.

### Multicenter immunoassay evaluation.

Contributing laboratories selected the assays they evaluated and the evaluation panels. All laboratories performed their evaluations in accordance with the Declaration of Helsinki. Informed consents were obtained, or other procedures required by their local institutions regarding research to improve diagnostic procedures with the use of samples obtained for routine clinical diagnostics were followed. All evaluated assays as of 17 July 2020 and their details are depicted in Table 1.

**TABLE 1:** Evaluated tests^*a*^

Test	Manufacturer	Format of test	Regulatory status	Antigen
2019-nCoV IgG/IgM rapid test cassette	Acro Biotech, Inc.	POCT	CE-IVD	Unknown
AFIAS COVID-19 Ab, IgM/IgG	Boditech Med. Inc.	POCT/AA	CE-IVD	Unknown
Architect SARS-CoV-2 IgG assay	Abott Core Laboratory	AA	CE-IVD	N
COVID-19 IgG/IgM Rapid test	Biomerica Inc.	POCT	CE-IVD	N
COVID-19 BSS	Biosynex SA	POCT	CE-IVD	Unknown
Corona virus COVID rapid test	Biozek medical	POCT	CE-IVD	Unknown
qSARS-CoV-2 IgG/IgM cassette rapid test	Cellex Inc.	POCT	CE-IVD	N, S
COVID-19 VIRCLIA IgG monotest	Vircell S.L.	AA	CE-IVD	Unknown
COVID-19 VIRCLIA IgM+IgA monotest	Vircell S.L.	AA	CE-IVD	Unknown
SARS-CoV-2 IgG ELISA kit	Creative Diagnostics	ELISA	RUO	Whole virus lysate antigen
SARS-CoV-2 IgM ELISA kit	Creative Diagnostics	ELISA	RUO	Unknown
DiagnoSure COVID-19 IgG/IgM rapid test cassette	GritOverseas Pte. Ltd	POCT	Unknown	Unknown
2019 nCOV IgG/IgM rapid test	Dynamiker Biotechnology (Tianjin) Co., Ltd	POCT	CE-IVD	N
Novel coronavirus COVID-19 ELISA IgG	Epitope Diagnostics, Inc.	ELISA	CE-IVD	Unknown
Novel coronavirus COVID-19 ELISA IgM	Epitope Diagnostics, Inc.	ELISA	CE-IVD	Unknown
Elecsys Anti-SARS-CoV-2	Roche Diagnostics Inc.	AA	CE-IVD	Recombinant N
SARS-CoV-2 IgG (protein S1)	Euroimmun AG	ELISA	CE-IVD	S1
SARS-CoV-2 IgA	Euroimmun AG	ELISA	CE-IVD	S1
Rapid SARS-CoV-2 antibody (IgM/IgG) test	InTec Products Inc.	POCT	CE-IVD	N
Liaison SARS-CoV-2 S1/S2 IgG	DiaSorin SpA	AA	CE-IVD	S1, S2
Maglumi 2019-nCoV IgG (CLIA)	Snibe Co. Ltd	AA	CE-IVD	Unknown
Maglumi 2019-nCoV IgM (CLIA)	Snibe Co. Ltd	AA	CE-IVD	Unknown
COVID-19 rapid test	Medea Medical Co.	POCT	Unknown	Unknown
NovaLisa SARS-CoV-2 IgG	NovaTec Immundiagnostica GmbH	ELISA	CE-IVD	Unknown
NovaLisa SARS-CoV-2 IgM	NovaTec Immundiagnostica GmbH	ELISA	CE-IVD	Unknown
NovaLisa SARS-CoV-2 IgA	NovaTec Immundiagnostica GmbH	ELISA	CE-IVD	Unknown
OnSite COVID-19 IgG/IgM rapid test	CTK Biotech, Inc.	POCT	CE-IVD	Unknown
Platelia SARS-CoV-2 Total Ab	Bio-Rad Laboratories Inc.	ELISA	CE-IVD	N
COVID-19 IgG/IgM rapid test	PRIMA Lab S.A.	POCT	CE-IVD	Unknown
2019-nCoV IgG/IgM test cassette	Prometheus Bio Inc.	POCT	CE-IVD	Unknown
recomWell SARS-CoV-2 IgG ELISA	Mikrogen GmbH	ELISA	CE-IVD	Recombinant N
Diagnostic kit for antibody IgM/IgG of novel coronavirus COVID-19	Shanghai LiangRun, Biomedicine Tech. Co., Ltd	POCT	CE-IVD	N, S
SARS-CoV-2 total	Siemens Healthineers	AA	CE-IVD	Unknown
COVID-19 coronavirus rapid test cassette	SureScreen Diagnostics	POCT	CE-IVD	Unknown
The non-invasive MEGA test of SARS-CoV-2	Absea Biotechnology Ltd	POCT	In development	Unknown
VIDAS anti-SARS-CoV-2 IgG	bioMérieux	AA	CE-IVD	Unknown
VIDAS anti-SARS-CoV-2 IgM	bioMérieux	AA	CE-IVD	Unknown
COVID-19 ELISA IgG	Vircell S.L.	ELISA	CE-IVD	N, S
COVID-19 ELISA IgM+IgA	Vircell S.L.	ELISA	CE-IVD	N, S
COVID-19 IgG/IgM rapid test cassette	Vomed diagnostics	POCT	Unknown	Unknown
Wondfo SARS-CoV-2 antibody test (LF method)	Guangzhou Wondfo Biotech Co., Ltd	POCT	CE-IVD	Unknown
VivaDiag COVID-19 IgM/IgG rapid test	VivaChek Biotech (Hangzhou) Co. Ltd.	POCT	CE-IVD	Unknown
Wantai SARS-CoV-2 Ab rapid test	Beijing Wantai Biological Pharmacy Enterprise Co., Ltd	POCT	Australia TGA	RBD
Wantai SARS-CoV-2 Ab ELISA	Beijing Wantai Biological Pharmacy Enterprise Co., Ltd	ELISA	CE-IVD	RBD
Wantai SARS-CoV-2 IgM ELISA	Beijing Wantai Biological Pharmacy Enterprise Co., Ltd	ELISA	CE-IVD	Unknown
2019-nCoV IgM/IgM combo test	Xiamen Boson Biotech Co., Ltd	POCT	CE-IVD	Unknown
COVID-19 IgG/IgM rapid test cassette	Zhejiang Orient Gene Biotech Co., Ltd./Healgen scientific LLC.	POCT	CE-IVD	N, S

a POCT, point-of-care test; AA, autoanalyzer; CE-IVD, Conformité Européenne-In Vitro Diagnostica; RUO, research use only; N, nucleocapsid protein; S, spike protein; RBD, receptor binding domain of S1 protein.

For determination of the sensitivity of the immunoassays, samples were used from reverse transcription-PCR (RT-PCR)-confirmed COVID-19 patients from all age groups, although they were predominantly from adults (≥19 years old). Data on sensitivity were aggregated and stratified by severity of infection and timing of sample collection, i.e., before or after 14 days post-onset of symptoms (DPO). Hospitalized COVID-19 cases were classified as severe cases and nonhospitalized cases as mild. If disease severity or DPO was not known, samples were excluded. For determination of the specificity, both population samples collected before December 2019 and samples from syndromic patients with respiratory infections with potentially cross-reactive microorganisms, e.g., common coronaviruses, were included. Samples negative for SARS-CoV-2 using RT-PCR, which were obtained during the pandemic, were excluded. Equivocal results were considered positive in sensitivity as well as in specificity cohorts. The aggregated results from 41 laboratories of sensitivity and specificity, including the 95% confidence interval (CI) based on Wilson score (13), were reported here.

For individual patient diagnostics, the predefined performance criteria for IgM and IgG antibodies, for both separately, were >95% sensitivity and >98% specificity if samples were obtained after 14 DPO. The same performance criteria were posed for epidemiological and serological prevalence studies but only for IgG antibodies. These predefined performance criteria are not absolute but were recommendations from the Serology Taskforce based on expert opinion and also used by other European member states (14). However, the applicability of these criteria will have to be continuously assessed by local experts in each specific context of use.

Additional to determining the sensitivity of the immunoassays with RT-PCR as a reference, three laboratories determined test sensitivity with a virus neutralization test (VNT; 50% plaque reduction/neutralization titer [PRNT_50_]) as a reference test, and these results were aggregated and reported here.

### Evaluation of nationwide collaborative approach.

Early July 2020, the added value of the collaborative national laboratory response was assessed with a short online questionnaire sent out through the NVMM and made using the online tool Typeform.

This survey consisted of questions about (i) the already implemented immunoassays, (ii) information sources that were used for the assay selection, (iii) if this nationwide collaborative approach was considered valuable and contributing to an increase of efficiency in laboratory response, (iv) if laboratories would be interested in a similar approach for future infectious disease crises, and (v) if there were other laboratory-related activities that should be nationally coordinated during a next public health crisis. The survey consisted of binary yes/no questions, upon which more details were requested using free text. All free-text answers were categorized by the authors.

## RESULTS

### Multicenter immunoassay evaluation.

The aggregated results from 41 laboratories of sensitivity and specificity, including the 95% CI based on Wilson score (13), as of 15 July 2020, were reported in Tables 2 and 3. A total of 17 laboratories had submitted data on test accuracy for 22 point-of-care (POC) tests (Table 2) and 39 laboratories for 25 ELISA and autoanalyzer tests (Table 3).

**TABLE 2:** Aggregated results for sensitivity and specificity of various commercialized POC tests using RT-PCR as a reference^*a*^

Test	No. of labs^*b*^	Ig type	Sensitivity								Specificity IgG	
			Severe infection, sample collection at >14 DPO		Mild infection, sample collection at >14 DPO		Severe infection, sample collectio*n* ≤14 DPO		Mild infection, sample collectio*n* ≤14 DPO		Prepandemic and cross-reactive samples	
			No. of samples^*c*^	% (95% CI)	No. of samples^*c*^	% (95% CI)	No. of samples^*c*^	% (95% CI)	No. of samples^*c*^	% (95% CI)	No. of samples^*d*^	% (95% CI)
Acro Biotech COVID-19 Rapid POC test	3	IgM	14/37	37.8 (24.1–53.9)	3/11	27.3 (9.7–56.6)	13/27	48.1 (30.7–66.0)	nd^*e*^	nd	48/50	96.0 (86.5–98.9)
		IgG	34/37	91.9 (78.7–97.2)	10/11	90.9 (62.3–98.4)	18/27	66.7 (47.8–81.4)	nd	nd	**49/50**	**98.0 (89.5**–**99.6)**
AFIAS COVID-19 Ab, IgM/IgG	1	IgM	0/7	0 (0–35.4)	0/36	0 (0–9.6)	0/4	0 (0–49.0)	0/2	0 (0–65.8)	**278/279**	**99.6 (98.0–99.9)**
		IgG	6/7	85.7 (48.7–97.4)	33/36	91.7 (78.2–97.1)	2/4	50.0 (15.0–85.0)	1/2	50.0 (9.5–90.5)	**274/279**	**98.2 (95.9–99.2)**
Biomerica COVID-19 IgG/IgM Rapid test	1	IgM	2/5	40.0 (11.8–76.9)	0/9	0 (0–29.9)	4/8	50.0 (21.5–78.5)	nd	nd	24/25	96.0 (80.5–99.3)
		IgG	5/5	100 (56.6–100)	7/9	77.8 (45.3–93.7)	5/8	62.5 (30.6–86.3)	nd	nd	**25/25**	**100 (86.7–100)**
Biosynex COVID–19 BSS	2	IgM	54/58	93.1 (83.6–97.3)	nd	nd	65/100	65.0 (55.3–73.6)	nd	nd	48/53	90.6 (79.7–95.9)
		IgG	**56/58**	**96.6 (88.3**–**99.0)**	nd	nd	44/100	44.0 (34.7–53.8)	nd	nd	**53/53**	**100 (93.2**–**100)**
Biozek Corona virus COVID rapid test	7	IgM	70/130	53.8 (45.3–62.2)	6/21	28.6 (13.8–50.0)	89/228	39.0 (32.9–45.5)	nd	nd	469/489	95.9 (93.8–97.3)
		IgG	**124/130**	**95.4 (90.3**–**97.9)**	18/21	85.7 (65.4–95.0)	133/228	58.3 (51.8–64.5)	nd	nd	**480/489**	**98.2 (96.5**–**99.0)**
Cellex qSARS-CoV-2 IgG/IgM cassette Rapid test	3	IgM	17/36	47.2 (32.0–63.0)	14/65	21.5 (13.3–33.0)	nd	nd	nd	nd	**111/112**	**99.1 (95.1**–**99.8)**
		IgG	**36/36**	**100 (90.4**–**100)**	49/65	75.4 (63.7–84.2)	nd	nd	nd	nd	**110/112**	**98.2 (93.7**–**99.5)**
		IgM/IgG^*f*^	0/2	0 (0–65.8)	nd	nd	53/90	58.9 (48.6–68.5)	nd	nd	nd	nd
DiagnoSure COVID-19 IgG/IgM rapid test cassette	1	IgM	5/5	100 (56.6–100)	5/10	50.0 (23.7–76.3)	4/8	50.0 (21.5–78.5)	nd	nd	**25/25**	**100 (86.7**–**100)**
		IgG	5/5	100 (56.6–100)	0/10	0 (0–27.8)	3/8	37.5 (13.7–69.4)	nd	nd	**25/25**	**100 (86.7**–**100)**
Dynamiker 2019 nCOV IgG/IgM Rapid test	2	IgM/IgG^*f*^	5/7	71.4 (35.9–91.8)	7/11	63.6 (35.4–84.8)	5/26	19.2 (8.5–37.9)	nd	nd	12/13	92.3 (66.7–98.6)
InTec Rapid SARS-CoV-2 antibody (IgM/IgG) Test	2	IgM	30/36	83.3 (68.1–92.1)	18/63	28.6 (18.9–40.7)	nd	nd	nd	nd	98/112	87.5 (80.1–92.4)
		IgG	**36/36**	**100 (90.4**–**100)**	43/63	68.3 (56.0–78.4)	nd	nd	nd	nd	107/112	95.5 (90.0–98.1)
		IgM/IgG^*f*^	nd	nd	nd	nd	65/76	85.5 (75.9–91.7)	nd	nd	nd	nd
Medea COVID-19 rapid test	1	IgM/IgG^*f*^	nd	nd	7/9	77.8 (45.3–93.7)	18/25	72.0 (52.4–85.7)	1/5	20.0 (3.6–62.4)	20/22	90.9 (72.2–97.5)
OnSite COVID-19 IgG/IgM rapid test	3	IgM/IgG^*f*^	0/2	0 (0–65.8)	22/42	52.4 (37.7–66.6)	7/18	38.9 (20.3–61.4)	7/18	38.9 (20.3–61.4)	77/81	95.1 (88.0–98.1)
Prima COVID-19 IgG/IgM rapid test	1	IgM/IgG^*f*^	0/2	0 (0–65.8)	nd	nd	3/18	16.7 (5.8–39.2)	nd	nd	nd	nd
Prometheus 2019-nCoV IgG/IgM test cassette	1	IgM/IgG^*f*^	0/2	0 (0–65.8)	nd	nd	4/18	22.2 (9.0–45.2)	nd	nd	nd	nd
Shanghai LiangRun, diagnostic kit for antibody IgM/IgG of novel coronavirus COVID-19	1	IgM	0/5	0 (0–43.4)	2/9	22.2 (6.3–54.7)	2/8	25.0 (7.1–59.1)	nd	nd	**25/25**	**100 (86.7**–**100)**
		IgG	5/5	100 (56.6–100)	3/9	33.3 (12.1–64.6)	4/8	50.0 (21.5–78.5)	nd	nd	**25/25**	**100 (86.7**–**100)**
SureScreen COVID-19 coronavirus rapid test cassette	1	IgM/IgG^*f*^	nd	nd	15/37	40.5 (26.3–56.6)	nd	nd	0/3	0 (0–56.1)	**55/56**	**98.2 (90.6**–**99.7)**
The non-invasive MEGA test of SARS-CoV-2	1	MEGA^*g*^ serum	4/4	100 (51.0–100)	nd	nd	nd	nd	nd	nd	0/5	0 (0–43.4)
		MEGA^*g*^ respiratory^*h*^	6/6	100 (61.0–100)	nd	nd	nd	nd	nd	nd	0/13	0 (0–22.8)
VivaDiag COVID-19 IgM/IgG rapid test	1	IgM/IgG^*f*^	8/8	100 (67.6–100)	1/1	100 (20.7–100)	nd	nd	nd	nd	**10/10**	**100 (72.2**–**100)**
Vomed COVID-19 IgG/IgM rapid test cassette	1	IgM/IgG^*f*^	**13/13**	**100 (77.2**–**100)**	6/8	75.0 (40.9–92.9)	5/10	50.0 (23.7–76.3)	0/4	0 (0–49.0)	**15/15**	**100 (79.6**–**100)**
Wantai SARS-CoV-2 Ab rapid test	2	IgM/IgG^*f*^	14/16	87.5 (64.0–96.5)	1/1	100 (20.7–100)	23/35	65.7 (49.2–79.2)	nd	nd	9/9	100 (70.1–100)
Wondfo SARS-CoV-2 antibody test (LF method)	1	IgM/IgG^*f*^	0/2	0 (0–65.8)	nd	nd	6/18	33.3 (16.3–56.3)	nd	nd	nd	nd
Xiamen BOSON 2019-nCoV IgM/IgM combo test	7	IgM	38/62	61.3 (48.8–72.4)	8/15	53.3 (30.1–75.2)	52/108	48.1 (39.0–57.5)	2/5	40.0 (11.8–76.9)	86/103	83.5 (75.1–89.4)
		IgG	**60/62**	**96.8 (89.0**–**99.1)**	10/15	66.7 (41.7–84.8)	54/108	50.0 (40.7–59.3)	1/5	20.0 (3.6–62.4)	97/103	94.2 (87.9–97.3)
		IgM/IgG^*f*^	6/8	75.0 (40.9–92.9)	nd	nd	24/35	68.6 (52.0–81.4)	nd	nd	nd	nd
Zhejiang Orient/Healgen COVID-19 IgM/IgM rapid test cassette	5	IgM	56/63	88.9 (78.8–94.5)	70/74	94.6 (86.9–97.9)	9/17	52.9 (31.0–73.8)	2/3	66.7 (20.8–93.9)	126/136	92.6 (87.0–96.0)
		IgG	**63/63**	**100 (94.3**–**100)**	69/74	93.2 (85.1–97.1)	8/17	47.1 (26.2–69.0)	2/3	66.7 (20.8–93.9)	**135/136**	**99.3 (96.0**–**99.9)**
		IgM/IgG^*f*^	19/21	90.5 (71.1–97.3)	7/9	77.8 (45.3–93.7)	95/158	60.1 (52.3–67.4)	1/4	25.0 (4.6–69.9)	71/73	97.3 (90.5–99.2)

a Values shaded in gray are data with <10 samples available. Bold values meet the predetermined criteria for patient diagnostics of >95% sensitivity and >98% specificity.

b Number of labs that shared evaluation data for this test.

c Positive samples/total evaluated samples.

d Negative samples/total evaluated samples.

e nd, no data available.

f IgM and IgG combined shared only.

g MEGA, IgM/IgE/IgG/IgA.

h Respiratory samples consist of E swab medium from nasopharyngeal, nose, or throat swabs.

**TABLE 3:** Aggregated results for sensitivity and specificity of various commercialized ELISA and autoanalyzer tests using RT-PCR as a reference^*a*^

Test	No. of labs^*b*^	Ig type	Sensitivity								Specificity IgG		
			Severe infection, sample collection at >14 DPO		Mild infection, sample collection at >14 DPO		Severe infection, sample collection at ≤14 DPO		Mild infection, sample collection at ≤14 DPO		Prepandemic and cross-reactive samples	
			No. of samples^*c*^	% (95% CI)	No. of samples^*c*^	% (95% CI)	No. of samples^*c*^	% (95% CI)	No. of samples^*c*^	% (95% CI)	No. of samples^*d*^	% (95% CI)
Architect SARS-CoV-2 IgG assay	7	IgG	110/117	94.0 (88.2–97.1)	74/83	89.2 (80.7–94.2)	46/127	36.2 (28.4–44.9)	1/13	7.7 (1.4–33.3)	**224/224**	**100 (98.9**–**100)**
COVID-19 VIRCLIA IgG monotest	3	IgG	39/45	86.7 (73.8–93.7)	**25/26**	**96.2 (81.1**–**99.3)**	50/87	57.5 (47.0–67.3)	nd^*e*^	nd	130/135	96.3 (91.6–98.4)
COVID-19 VIRCLIA IgM+IgA monotest	2	IgM+IgA	**30/31**	**96.8 (83.8**–**99.4)**	4/6	66.7 (30.0–90.3)	33/41	80.5 (66.0–89.8)	nd	nd	68/75	90.7 (82.0–95.4)
Creative Diagnostics SARS-CoV-2 IgG ELISA kit	1	IgG	18/24	75.0 (55.1–88.0)	24/46	52.2 (38.1–65.9)	9/32	28.1 (15.6–45.4)	nd	nd	**77/78**	**98.7 (93.1**–**99.8)**
Creative Diagnostics SARS-CoV-2 IgM ELISA kit	1	IgM	20/24	83.3 (64.1–93.3)	25/46	54.3 (38.1–65.9)	18/32	56.3 (39.3-71.8)	nd	nd	76/78	97.4 (91.1–99.3)
EDI novel coronavirus COVID-19 ELISA IgG	8	IgG	**94/97**	**96.9 (91.3**–**98.9)**	73/93	78.5 (69.1–85.6)	73/109	67.0 (57.7–75.1)	5/14	35.7 (16.3–61.2)	245/257	95.3 (92.0–97.3)
EDI novel coronavirus COVID-19 ELISA IgM	7	IgM	76/97	78.4 (69.2–85.4)	20/63	31.7 (21.6–44.0)	67/111	60.4 (51.1–69.0)	2/10	20.0 (5.7–51.0)	**218/221**	**98.6 (96.1**–**99.5)**
Elecsys anti-SARS-CoV-2	7	IgT	187/198	94.4 (90.3–96.9)	107/120	89.2 (82.3–93.6)	73/157	46.5 (38.9–54.3)	15/20	75.0 (53.1–88.8)	**471/472**	**99.8 (98.8**–**100)**
Euroimmun SARS-CoV-2 IgG (protein S1)	13	IgG	**220/229**	**96.1 (92.7**–**97.9)**	99/130	76.2 (68.1–82.7)	117/251	46.6 (40.5–52.8)	12/22	54.5 (34.7–73.1)	**641/652**	**98.3 (97.0**–**99.1)**
Euroimmun SARS-CoV-2 IgA	7	IgA	**95/99**	**96.0 (90.1**–**98.4)**	42/66	63.6 (51.6–74.2)	108/137	78.8 (71.3–84.8)	2/5	40.0 (11.8–76.9)	331/367	90.2 (86.7–92.8)
Liaison SARS-CoV-2 S1/S2 IgG	18	IgG	345/366	94.3 (91.4–96.2)	134/165	81.2 (74.6–86.4)	55/275	20.0 (15.7–25.1)	14/21	66.7 (45.4–82.8)	920/946	97.3 (96.0–98.1)
Maglumi 2019-nCoV IgG (CLIA)	1	IgG	**24/24**	**100 (86.2**–**100)**	19/22	86.4 (66.7–95.3)	1/1	100 (20.7–100)	10/12	83.3 (55.2–95.3)	60/62	96.8 (89.0–99.1)
Maglumi 2019-nCoV IgM (CLIA)	1	IgM	**23/24**	**95.8 (79.8**–**99.3)**	15/22	68.2 (47.3–83.6)	1/1	100 (20.7–100)	11/12	91.7 (64.6–98.5)	60/62	96.8 (89.0–99.1)
NovaLisa SARS-CoV-2 IgG	1	IgG	28/31	90.3 (75.1–96.7)	5/5	100 (56.6–100)	16/36	44.4 (29.5–60.4)	nd	nd	69/72	95.8 (88.5–98.6)
NovaLisa SARS-CoV-2 IgM	1	IgM	18/31	58.1 (40.8–73.6)	0/5	0 (0–43.4)	10/36	27.8 (15.8–44.0)	nd	nd	**71/72**	**98.6 (92.5**–**99.8)**
NovaLisa SARS-CoV-2 IgA	1	IgA	28/31	90.3 (75.1–96.7)	2/5	40.0 (11.8–76.9)	24/36	66.7 (50.3–79.8)	nd	nd	64/72	88.9 (79.6–94.3)
Platelia SARS-CoV-2 total Ab	3	IgT	25/27	92.6 (76.6–97.9)	74/83	89.2 (80.7–94.2)	45/72	62.5 (48.7–70.3)	2/3	66.7 (20.8–93.9)	115/122	94.3 (88.6–97.2)
recomWell SARS-CoV-2 IgG ELISA	8	IgG	**104/108**	**96.3 (90.9**–**98.6)**	79/91	86.8 (78.4–92.3)	62/95	65.3 (55.3–74.1)	9/30	30.0 (16.7–47.9)	318/330	96.4 (93.8–97.9)
Siemens SARS-CoV-2 total antibody	5	IgT	100/108	92.6 (86.1–96.2)	42/50	84.0 (71.5–91.7)	38/69	55.1 (43.4–66.2)	nd	nd	**183/185**	**98.9 (96.1**–**99.7)**
VIDAS anti-SARS-CoV-2 IgG	1	IgG	**22/22**	**100 (85.1**–**100)**	nd	nd	2/6	33.3 (9.7–70.0)	nd	nd	18/20	90.0 (69.9–97.2)
VIDAS anti-SARS-CoV-2 IgM	1	IgM	**22/22**	**100 (85.1**–**100)**	nd	nd	4/6	66.7 (30.0–90.3)	nd	nd	16/18	88.9 (67.2–96.9)
Vircell COVID-19 ELISA IgG	6	IgG	**88/91**	**96.7 (90.8**–**98.9)**	33/37	89.2 (75.3–95.7)	102/129	79.1 (71.3–85.2)	2/5	40.0 (11.8–76.9)	248/265	93.6 (90.0–96.0)
Vircell COVID-19 ELISA IgM+IgA	5	IgM+IgA	**88/91**	**96.7 (90.8**–**98.9)**	26/37	70.3 (54.2–82.5)	73/103	70.9 (61.5–78.8)	1/5	20.0 (3.6–62.4)	146/178	82.0 (75.7–87.0)
Wantai SARS-CoV-2 Ab ELISA	25	IgT	**630/646**	**97.5 (96.0**–**98.5)**	**355/372**	**95.4 (92.8**–**97.1)**	359/459	78.2 (74.2–81.7)	27/40	67.5 (52.0–79.9)	**1328/1334**	**99.6 (99.0**–**99.8)**
Wantai SARS-CoV-2 IgM ELISA	11	IgM	139/149	93.3 (88.1–96.3)	64/81	79.0 (68.9–86.5)	123/166	74.1 (66.9–80.2)	10/24	41.7 (24.5–61.2)	**372/375**	**99.2 (97.7**–**99.7)**

a Values shaded in gray are data with <10 samples available. Bold values meet the predetermined criteria for patient diagnostics of >95% sensitivity and >98% specificity.

b Number of labs that shared evaluation data for this test.

c Positive samples/total evaluated samples.

d Negative samples/total evaluated samples.

e nd, no data available.

The Biosynex, Biozek, Cellex, Vomed, and Zhejiang Orient/Healgen were the only POC tests that met the predetermined criteria of >95% sensitivity combined with 98% specificity for diagnostics in severe infections with samples taken after 14 DPO but only for IgG (or total Ig for Vomed). None of the POC tests complied to predetermined criteria for IgM only or for patients with mild or asymptomatic infections (Table 2).

Although multiple ELISA and autoanalyzer assays testing IgM antibodies met the predetermined criteria for sensitivity (>95%) in patients with severe infections with a sample collection after 14 DPO, none of them reached a specificity of >98%, and therefore, they did not fulfill all criteria (Table 3). For IgG or IgTotal targeted assays, only Euroimmun IgG and Wantai Ab ELISAs met both sensitivity and specificity criteria in severe infections if samples were taken after 14 DPO (Table 3). For diagnostics in mild or asymptomatic infections, only Wantai Ab met the predetermined criteria for use in diagnostics if samples were taken after 14 DPO (Table 3).

Additionally, the results of the sensitivity of the immunoassays if virus neutralization tests (VNTs; PRNT_50_) were used as reference instead of RT-PCR were reported in Table 4. We observed a good sensitivity (>95%) in severe infections if samples were taken after 14 DPO for the POC tests InTec and Zhejiang Orient/Healgen and for ELISAs Euroimmun IgG and IgA and Wantai Ab (Table 4). In mild infections, we observed a good sensitivity for Zhejiang Orient/Healgen rapid test and for the Wantai Ab ELISA if samples were taken after 14 DPO (Table 4).

**TABLE 4:** Aggregated results of sensitivity of various commercialized antibody tests, using virus neutralization tests as a reference^*a*^

Test	Reference neutralization test	Sensitivity (sample collection at >14 DPO)			
		Severe infection		Mild infection	
		No. of samples^*b*^	% (95% CI)	No. of samples^*b*^	% (95% CI)
Architect SARS-CoV-2 IgG assay	PRNT_50_	nd^*c*^	nd	9/13	69.2 (42.4−87.3)
Cellex qSARS-CoV-2 IgG/IgM cassette rapid test	PRNT_50_	66/74	89.2 (80.1–94.4)	48/57	84.2 (72.6–91.5)
InTec rapid SARS-CoV-2 antibody (IgM/IgG) test	PRNT_50_	74/76	97.4 (90.9–99.3)	41/55	74.5 (61.7–84.2)
Euroimmun SARS-CoV-2 IgG (protein S1)	PRNT_50_	26/27	96.3 (81.7–99.3)	1/3	33.3 (6.1–79.2)
	VNT50%	34/35	97.1 (85.5–99.5)	nd	nd
	VNT90%	14/14	100 (78.5–100)	nd	nd
Euroimmun SARS-CoV-2 IgA	PRNT_50_	27/27	100 (87.5–100)	3/3	100 (43.9–100)
Liaison SARS-CoV-2 S1/S2 IgG	PRNT_50_	95/104	91.3 (84.4–95.4)	46/55	83.6 (71.7–91.1)
Wantai SARS-CoV-2 Ab ELISA	PRNT_50_	118/118	100 (96.8–100)	75/77	97.4 (91.0–99.3)
	VNT50%	155/155	100 (97.6–100)	nd	nd
	VNT90%	153/155	98.7 (95.4–99.6)	nd	nd
Wantai SARS-CoV-2 IgM ELISA	PRNT_50_	101/116	87.1 (79.8–92.0)	65/75	86.7 (77.2–92.6)
Zhejiang Orient/Healgen COVID-19 IgM/IgG rapid test cassette	PRNT_50_	155/155	100 (97.6–100)	56/56	98.7 (95.4–99.6)

a Values shaded in gray are data with <10 samples available.

b Positive samples/total evaluated samples.

c nd, no data available.

### Evaluation of nationwide collaborative laboratory response.

**FIG 2:**
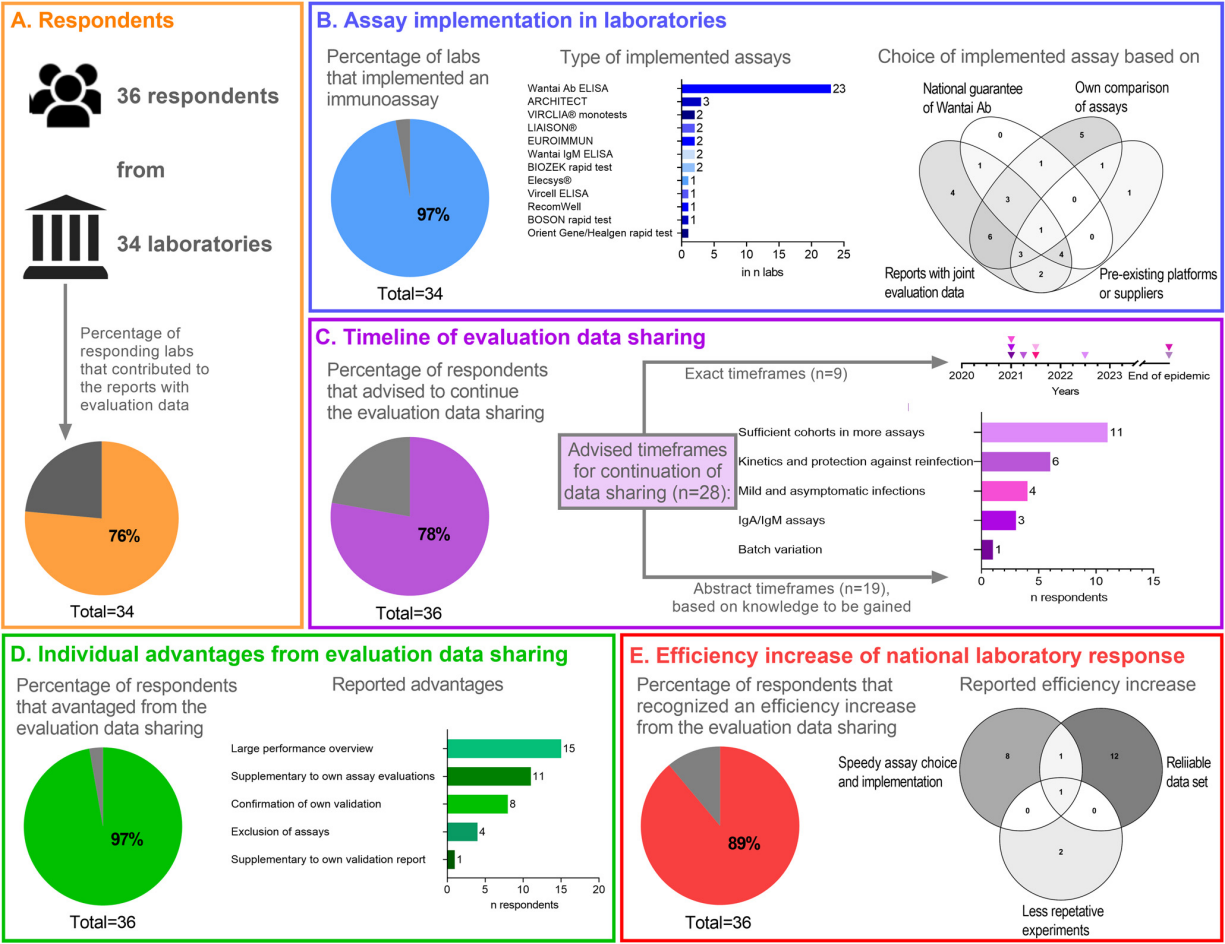
Summary of responses to the survey on the added value of the joint collection and sharing of assay evaluation data across laboratories in the Netherlands.

To assess how the joint collection and sharing of evaluation data of commercial immunoassays were perceived and whether they contributed to an improved laboratory response in the Netherlands, we sent out a short survey to the Dutch COVID-19 diagnostic laboratories. In total, 36 representatives from 34 of approximately 50 registered medical microbiological laboratories (60% to 70%) in the Netherlands responded to the survey (Fig. 2A). The results of the survey were summarized in Fig. 2.

Almost all laboratories (33/34, 97%) had implemented a serological assay for the detection of antibodies against SARS-CoV-2 (Fig. 2B). Most of them (80%) implemented at least one ELISA test. The Wantai SARS-CoV-2 Ab ELISA was implemented by the majority of laboratories (*n* = 23). The choice for the implemented test was for 24 (71%) laboratories based on the reports with shared evaluation data published by the Serology Taskforce. For four of them, these reports were the sole source on which they based their choice (Fig. 2B). Other reported information sources were comparisons of tests in their own laboratory (*n* = 20), the already local existing platforms and/or relationships with suppliers (*n* = 12), a literature review (*n* = 1), and the guarantee of the national stock of Wantai SARS-CoV-2 Ab ELISA (*n* = 10) (Fig. 2B).

A total of 28 (76%) of the respondents advised to continue the data sharing (Fig. 2C). Additionally, they provided the time frame for this continuation, 9 (32%) gave an exact frame with end date, and 19 (68%) gave an abstract time frame, based on knowledge that still needs to be gained (Fig. 2C). All but one of the respondents (97%) indicated that their laboratory benefitted from the joint collection and sharing of evaluation data, and the specific advantages were specified (Fig. 2D). Next to local added value, 89% (32 of 36) of the respondents thought that the (almost) real-time sharing of data increased the efficiency of the national laboratory response regarding serological testing for SARS-CoV-2 (Fig. 2E). Experiences were that it enabled laboratories to make a more rapid choice and quickly implement tests in a confusing and aggressive market (*n* = 10), that more data are at their disposal which results in more robust evaluations (*n* = 14), and that it avoided repetitive experiments in multiple laboratories and a subsequent waste of budget (*n* = 3).

All respondents considered that a similar approach should be employed during future epidemics. In total, 32 (89%) respondents reported that other activities, besides the sharing of evaluation data, should be coordinated at a national level in a future epidemic. Suggestions included providing standard sample panels or high-quality biobanking at the national level (*n* = 18); organizing external quality assessment panels (*n* = 5); providing and distributing material and reagents (*n* = 3); joint purchasing of assays, material, and reagents (*n* = 3); creating a consensus about the role and meaning of serology (*n* = 2); organizing interlaboratory communication, i.e., through webinars (*n* = 3); and performing one central evaluation of all tests, enabling local verification only (*n* = 2).

## DISCUSSION

Upon the emergence of SARS-CoV-2 as a novel pathogen with pandemic spread, the diagnostic market was overflowing with assays for molecular detection; assays for detection of SARS-CoV-2-specific IgG, IgM, and/or IgA; and antigen tests. All medical laboratories need to validate any new assay before implementation for diagnostic purposes as part of their quality management system ISO 15189 (10). In a nonpandemic context, each laboratory individually evaluates and validates diagnostic tests for their own implementation, leading to dispersed and nonaccessible data of valuable assay performance. However, the rapid pandemic spread of a novel pathogen required a different, collaborative laboratory response, when sufficient test kits and properly defined evaluation panels are initially lacking, to collect a robust quantity of test performance data within the short time frame that is needed for an adequate response.

Already in the early phase of the outbreak, the first immunoassay evaluation data were shared by a few laboratories which prompted the Dutch government to establish a large stockpile of the Wantai SARS-CoV-2 Ab ELISA to guarantee availability for Dutch laboratories. In the following weeks, 41 laboratories joined forces and shared their ongoing evaluations on a weekly basis to enable the compilation of larger data sets for multiple serological tests that exceeded the capacity of each individual laboratory. The shared evaluation data were summarized and updated in reports that came out regularly and were made publicly available. These reports produced a complete overview of the performances of various serological tests at the service of all laboratories and policymaking institutes, instead of the otherwise valuable but less integrated information that one laboratory independently can yield.

When using RT-PCR as a reference test, the aggregated data of the POC evaluations showed that five of the investigated POC antibody tests met the predetermined criteria for IgG or Ig total diagnostics in severe infections, where samples were collected after 14 DPO. However, results for 4 POC tests were based on fewer than 100 samples. Additionally, two ELISAs met the predetermined criteria for IgG or Ig total diagnostics in this patient group based on a sufficient amount of samples. However, currently, in practice, the relevance and added value of serology-based diagnostics compared with other diagnostic methods that aim to directly detect the presence of virus seem to focus on patients with a negative SARS-CoV-2 RT-PCR and a persistent strong suspicion for COVID-19. Indeed, this added value was clearly demonstrated in the SARS-COV-2 20C/H655Y hospital cluster in Brittany, France, in March 2021 where cases were confirmed based on serology, while RT-PCR on nasopharyngeal swabs failed (15). None of the 22 investigated POC antibody tests met the predetermined criteria for IgM and IgG sensitivity and IgG specificity for use in patients with mild or asymptomatic infections if based on a sufficient amount of diagnostic samples. The only test that met the criteria in the patient group with mild or asymptomatic SARS-CoV-2 infections is the Wantai SARS-CoV-2 Ab ELISA, which is based on the detection of total antibodies. None of the immunoassays that detect only IgM or IgA met the diagnostic criteria, indicating that they are not suitable for the diagnosis of acute infections. These data underline the importance of extensive validation in the right (sub)populations and settings. Before such an extensive validation, it is not appropriate to use (rapid) immunoassays for clinical decision making to guide dedicated measures for specific subpopulations and to guide general control measures.

Primarily, positive nucleic acid amplification testing (NAT) prior to sample collection for the use in immunoassays was used as a reference for sensitivity calculation. Because of the kinetics of an infection, PCR will be positive only in the acute stage, followed by IgM antibody production that wanes relatively fast, and IgG that will be detectable much longer (16). To limit the possibility of premature sample collection for antibody detection after positive SARS-CoV-2 PCR, only sensitivity measured in serological samples that were taken >14 DPO was considered reliable. However, even so, it cannot be completely ruled out that some confirmed patients did not develop any detectable immune response, thereby inflating the sensitivity (17). This could also partly explain the lower sensitivity in patient populations with mild infection, as their immune response is less intense than that of severe infections (18–20). The use of virus neutralization as a reference test aimed at determining the relationship between the outcomes of the routine serological assays and the presence of functional antibodies, i.e., neutralizing antibodies. However, this comparison was performed by only three laboratories in the country and yielded a data set that was too limited to draw firm conclusions.

Limitations of this study were that laboratories used their own protocols. Ideally, a joint national laboratory response is based on one common, standardized protocol for assay evaluation shared among the laboratories at the start of the outbreak. Use of a standardized protocol would greatly enhance the comparability between studies. Another limitation is that for some immunoassays, evaluation data from only a small amount of samples were available. This limitation was due to (i) the fact that laboratories chose their own assays to evaluate and (ii) the period in the pandemic that certain assays became available, as some assays were scarce and/or laboratories had difficulty accessing positive sample material for mildly infected or asymptomatic patients. Because of these scarcities, a power analysis could not be made before the study started, and all available samples and evaluation data were welcomed. Based on expert opinion, the results of test evaluations were considered reliable if at least 100 samples were used, as the range of the 95% confidence interval width decreased with these sample numbers. The difficulty accessing positive sample material can be solved by, depending on the outbreak at hand, providing well-documented reference materials from a (virtual) national biobank.

The evaluation of this specific activity of the national laboratory response to the emergence of SARS-CoV-2 showed that the collaborative effort was highly appreciated and directly informed decision making on implementation of diagnostic tests by individual laboratories. The consensus among the survey respondents was that the joint assay evaluation had increased the overall efficiency of the Dutch laboratory response. By sharing the weekly reports with the ECDC and WHO, the Dutch laboratories contributed to the EU and worldwide laboratory response to SARS-CoV-2 (8, 14).

To conclude, the shared data generated by the joint approach in the Netherlands increased the efficiency of a nationwide laboratory response. First, it quickly confirmed the advice in the scientific brief from 8 April 2020 of the WHO that POC immunoassays should be used only for research purposes because of questionable performances (21). Second, there were concerns about quick implementation of immunoassays with unproven performance characteristics because there is considerable pressure on laboratories from the public and governments (22). Our data quickly gave laboratories an overview of the initial performances of the assays and enabled them to make a more evidence-based choice for quality assays. Third, these joint forces had a positive influence on the number of assays and samples that could be processed, despite the initial shortage of tests and samples from a variety of patients cohorts. Finally, this approach enabled laboratories to verify tests rather than evaluate and validate it extensively, preventing duplicate experiments and waste of budget in health care settings.

Many laboratories, Dutch governmental institutes, and public health institutes endorsed the value of the collaborative evaluations of immunoassays described here; therefore, this approach in which evaluation data are shared will be continued in the upcoming period. Currently, the international market of SARS-CoV-2 diagnostics also focuses on antigen tests, and the same collaborative approach is employed for these assays in the Netherlands.
